# Molecular Characterization of a Novel Monopartite Begomovirus Infecting Weeds and Important Crops in Yunnan, China

**DOI:** 10.3390/v18080876

**Published:** 2026-08-11

**Authors:** Liling Zhao, Jing Zhong, Shuiying Zhang, Tingting Li, Yueyan Yin, Ru Dong, Ming Ding

**Affiliations:** Key Laboratory of Agricultural Biotechnology of Yunnan Province, Institute of Biotechnology and Germplasm Resources, Yunnan Academy of Agricultural Sciences, No. 2238 Beijing Road, Panlong District, Kunming 650205, China; zhaolilingyunnan@163.com (L.Z.); zhong_jing1228@163.com (J.Z.); shuiying8891@163.com (S.Z.); litingting9618@163.com (T.L.); yinyy0324@163.com (Y.Y.); rud_bio@163.com (R.D.)

**Keywords:** *Begomovirus*, novel, weed, crop, recombination

## Abstract

The genus *Begomovirus* constitutes a group of devastating plant viruses causing significant economic losses in the production of agricultural crops. In the present study, conducted in Yunnan province, China, a novel monopartite begomovirus was identified from *Bidens pilosa*, *Crassocephalum crepidioides*, tomato, pepper, and common bean, showing severe disease symptoms. The complete genome of the virus shows the typical organization of monopartite begomoviruses and shares the highest nucleotide sequence identity (88.73–88.95%) with crassocephalum yellow vein virus (CraYVV). According to the species criteria of the genus *Begomovirus*, this virus is a novel Begomovirus species, which has since been named “bidens pilosa leaf crumple virus (BpLCrV)”. Recombination analysis revealed that the novel species is a potential recombinant begomovirus derived from CraYVV and ageratum leaf curl virus (ALCuV), and phylogenetic analysis showed that BpLCrV was clustered with tomato yellow leaf curl Thailand virus (TYLCTHV) from China. We successfully developed a BpLCrV infectious clone. Agrobacterium-mediated inoculation of the BpLCrV infectious clone could effectively infect *Nicotiana benthamiana*, *Nicotiana glutinosa*, and *Datura stramonium* and cause disease symptoms. Thus, in this study, BpLCrV was, for the first time, identified and characterized as a novel begomovirus that infects not only weeds but also different important crops, potentially threatening agricultural production.

## 1. Introduction

*Begomovirus*, a class of plant viruses with circular, single-stranded DNA genomes, causes significant economic losses in the production of vegetable, root, and fiber crops worldwide [1]. At present, it is the largest genus in the virus kingdom, composed of 445 virus species recognized by the International Committee on Taxonomy of Viruses (ICTV) [2]. Based on their genome components, begomoviruses can be divided into bipartite (consisting of DNA-A and DNA-B components) and monopartite (consisting of only one component similar to DNA-A of bipartite viruses). The monopartite genome DNA-A is about 2.5–3.0 kb and encompasses seven ORFs, including V1 and V2 in sense sequence and C1, C2, C3, C4, and C5 in complementary sense sequence [3]. These ORFs encode proteins functioning in viral replication (C1 and C3), transcription (C2), virion assembly (V1), and suppression gene silencing (V2, C2, C4, C5) [4].

Weeds play an important role in the epidemiology of begomoviruses. Many weed plants have been reported as natural hosts of begomoviruses, and the majority of these weed plants belong to the families *Asteraceae*, *Malvaceae*, *Amaranthaceae*, *Euphorbiaceae*, *Solanaceae*, *and Leguminosae* [4,5,6,7,8,9]. Meanwhile, weeds also serve as “melting pots” for the evolution of new viral species and strains [10], such as novel begomoviruses of sida yellow mosaic Gujarat virus [11], chenopodium leaf distortion virus [12], and macroptilium bright yellow interveinal virus [13].

Nevertheless, most weed begomoviruses often fail to infect crops under field conditions, though it is supposed that these viruses can be spilled over from original hosts (wild plants) to new hosts (crops) and transmitted back from crops to wild plant hosts [14]. Interestingly, it is known that tomato yellow leaf curl virus (TYLCV), a global geminivirus, has a very diverse host range, including not only 49 plant species under experimental conditions [15] but also 25 plant species in field conditions [4]. Here, we identified and characterized a novel pathogenic monopartite begomovirus that can infect weed species of *Bidens pilosa* and *Crassocephalum crepidioides*, as well as crop species of tomato, common bean, and pepper in field conditions, showing potential threat to the production of important agricultural crops.

## 2. Materials and Methods

### 2.1. Sample Collection and Virus Detection

A field survey of begomovirus diversity was conducted in Yunnan Province from 2017 to 2018 and approximately one thousand samples were collected from different growth fields. Total DNA was extracted from these samples using the CTAB method [16]. Initially, PCR was conducted with the degenerated primers PA/PB (for DNA-A), PCRc1/PBL1v2040 (for DNA-B), β01/β02 (for betasatellite), and UNA101/102 (for alphasatellite) to detect begomovirus and satellite infection [17,18,19,20]. The PCR products were analyzed on 1% agarose gel and the desired band was purified using a gel extraction kit (Axygen, Union City, CA, USA), then subsequently ligated into the pGEM-T easy vector (Promega Corporation, Madison, WI, USA). The ligation mixture was used to transform *Escherichia coli* strain DH5*α*. Positive clones were sequenced at commercial facilities of Life Technologies, Shanghai, China.

### 2.2. Cloning, Sequencing, and Sequence Analysis

Based on the nucleotide sequence of the obtained fragments, specific primer pairs (F: CCACTCCCGCATCCAAGGTG, R: GGAAATGACTATATCGGCGG) were designed for amplification of the full-length begomovirus DNA from the total DNA. The PCR conditions were as follows: initial denaturation at 98 °C for 2 min; 35 cycles of denaturation at 98 °C for 10 s, annealing at 59 °C for 30 s, and extension at 68 °C for 3.0 min; and a final extension at 68 °C for 10 min. The PCR products were analyzed on 1% agarose gel and the desired band was purified using a gel extraction kit (Axygen, Union City, CA, USA) and subsequently ligated into the pGEM-T easy vector (Promega Corporation, Madison, WI, USA). The ligation mixture was used to transform *Escherichia coli* strain DH5*α*. Positive clones were sequenced at commercial facilities of Life Technologies, Shanghai, China. Open reading frames (ORFs) of the nucleotide sequences were identified by DNAMAN Version 7 (Lynnon Biosoft, Quebec, QC, Canada). Similarity alignment was performed using the program SDT version 1.3 [21] or using BLASTn (BLAST version 2.14.0, NCBI, Bethesda, MD, USA) in the NCBI nucleotide database. The sequences used for SDT analysis are listed in Table S6.

### 2.3. Phylogenetic Reconstruction and Recombination Analysis

Phylogenetic analysis was performed using the Maximum Likelihood (ML) method in MEGA X based on the GTR + G + I model, with bootstrap support evaluated using 1000 replicates. The analysis included 37 nucleotide sequences listed in Table S6. Recombination was analyzed with Recombination Detection Program 4, using the RDP, GENECONV, BootScan, MaxChi, Chimaera, SiScan, and 3Seq methods. The sequences used for recombination analysis are listed in Table S6.

### 2.4. Construction of the Infectious Clone and Agrobacterium-Mediated Inoculation

To construct the infectious clones of BpLCrV, 1.5 mer tandem repeats of DNA A were cloned into the plant binary vector pBinPLUS. The recombinant plasmids were then introduced into *Agrobacterium tumefaciens* strain EHA105 via electroporation. The full-length genome of YN6330-2 was amplified using specific primer pairs YN6330FL-BF/BR (BF: CGGGATCCATTATTAAATGAGTTTCCTG, BR: CGGGATCCCACATAGTGCGGAGTGCA), and cloned into pGEM-T Easy Vector to obtain pGEM-T-YN6330-2-1A. Meanwhile, the PCR fragment (1.37 kb) of YN6330-2, including the IR of the virus, was amplified using specific primer pairs YN6330FL-BF and YN6330PL-SalI-R (YN6330PL-SalI-R: GCGTCGACGTTTGTGACGAGGACAGTGGG), and cloned into pGEM-T Easy Vector to obtain pGEM-T-YN6330-2-0.5A. After sequencing, the pGEM-T-YN6330-2-0.5A was digested with EcoRI and SalI restriction enzymes (Thermo Fisher Scientific, Waltham, MA, USA) and cloned into the binary vector pBinPLUS to obtain pBinPLUS-YN6330-2-0.5A. The full-length YN6330-2 was digested with EcoRI from the pGEM-T-YN6330-2-1A and cloned into the pBinPLUS-YN6330-2-0.5A to produce pBinPLUS-YN6330-2-1.5A, containing a head-to-tail 1.5 dimer of the BpLCrV genome. Subsequently, the recombinant plasmid pBinPLUS-YN6330-2-1.5A was transformed into *A. tumefaciens* strain EHA105 to produce an infectious clone of YN6330-2. Agroinfiltration of *Nicotiana benthamiana* (*N. benthamiana*), *Nicotiana glutinosa* (*N. glutinosa*), tomato (*Solanum lycopersicum*, *S. lycopersicum*), pepper (*Capsicum annuum*, *C. annuum*), and *Datura stramonium* (*D. stramonium*) plants were performed as previously described [22].

## 3. Results

### 3.1. Symptoms and Virus Detection

**Figure 1:**
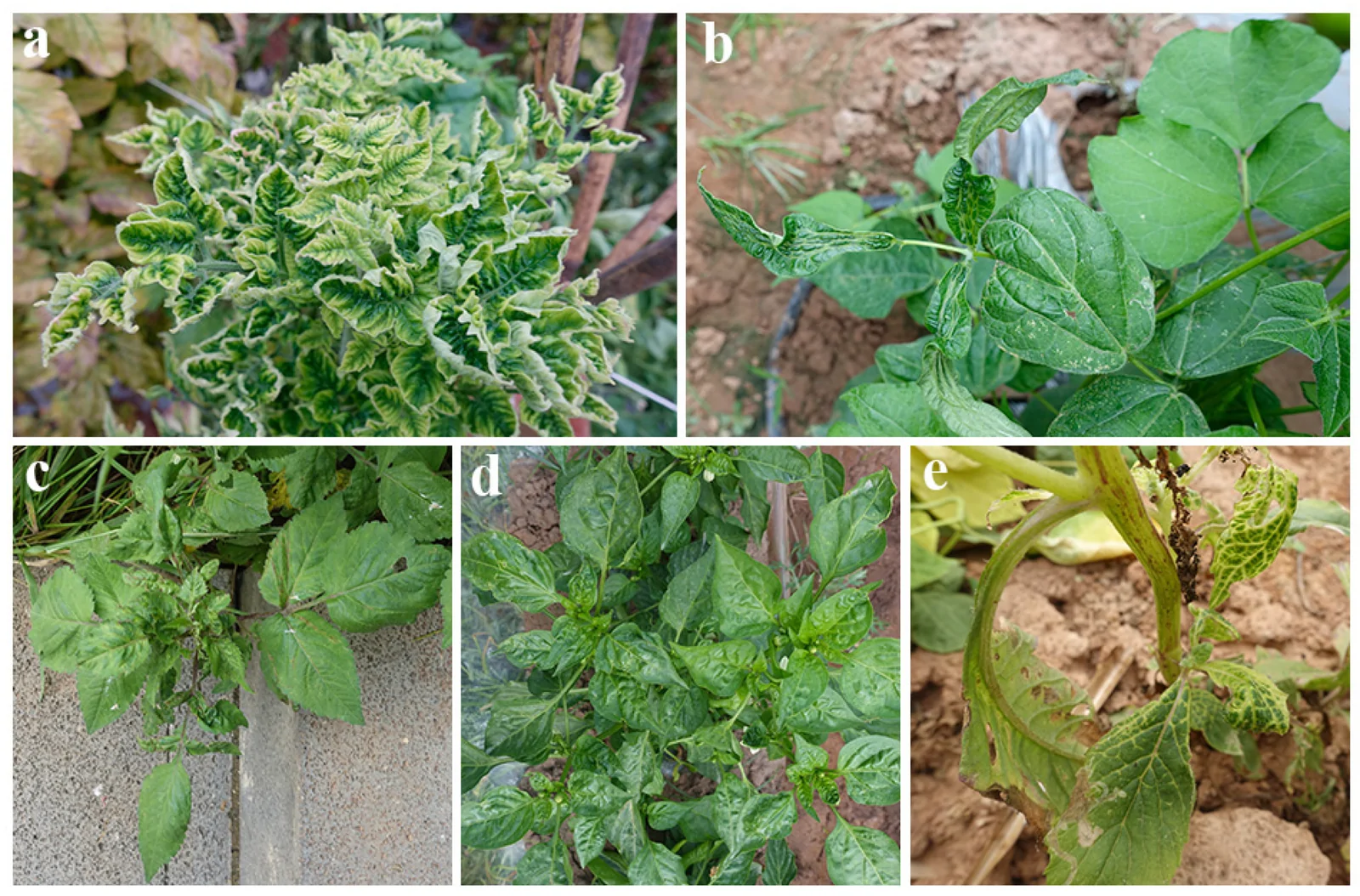
Begomovirus-like symptoms on tomato (**a**), common bean (**b**), *Bidens pilosa* (**c**), pepper (**d**), and *Crassocephalum crepidioides* (**e**) plants collected under field conditions.

During the field survey in Yunnan, we obtained over 200 samples from plants with typical symptoms induced by begomovirus, such as leaf curling, stunted growth, vein yellowing, and enation. Among them, leaf curl disease was first observed in *Bidens pilosa* with about 5% disease incidence in Honghe, Yunnan (Figure 1c). Meanwhile, seven leaf samples were also collected from tomato, common bean (*Phaseolus vulgaris*), *Bidens pilosa*, pepper, and *Crassocephalum crepidioides* plants exhibiting typical symptoms of begomovirus infection (Figure 1, Table S1). An approximately 500 bp DNA fragment was amplified from these seven leaf samples and a total of 14 positive clones were subjected to sequencing. In addition, an approximately 1.3 kb DNA fragment was amplified from two tomato and one common bean sample (Figure S1) and a total of six positive clones were subjected to sequencing. In contrast, all samples tested negative for DNA-B and alphasatellite components using degenerated primer sets (Figure S1).

Sequence comparison showed that all 14 PA/PB fragment sequences shared 91.5% identity with the *CP* gene of tomato yellow leaf curl Vietnam virus (TYLCVV). Furthermore, five sequences of betasatellite fragments were obtained. Sequence comparison showed that three sequences shared more than 98% identity with tomato leaf curl China betasatellite isolate 24YN1343-3B (PV938431), and two sequences shared more than 93% identity with malvastrum yellow vein Yunnan betasatellite (GU199589 and AM236778). These five betasatellite sequences have been submitted to GenBank under the accession numbers given in Table S2. All sequences showed a genome organization typical of betasatellites, comprising a single conserved coding sequence (the *βC1* gene), a satellite conserved region (SCR), and an adenine-rich (A-rich) region (Table S2).

### 3.2. Cloning, Sequencing and Sequence Analysis

A DNA-A fragment of the accession was amplified through PCR and a total of 12 sequences were obtained. These 12 sequences of DNA-A consisting of 2735 nucleotides have been submitted to GenBank under the accession numbers given in Table S3. All sequences showed a genome organization typical of monopartite begomovirus reported from the Old World: two virus-sense genes encoding the capsid protein (CP/V1) and V2 protein (V2) and four complementary-sense genes encoding the replication-associated protein (Rep/C1), a transcriptional activator protein (TrAP/C2), a replication enhancer protein (REn/C3), and the C4 protein (C4) (Table S3). All these sequences also have an intergenic region (IR), which includes the stem-loop structure nucleotide sequence TAATATT↓AC, TATA box, and iteron sequences (GGTGT).

**Figure 2:**
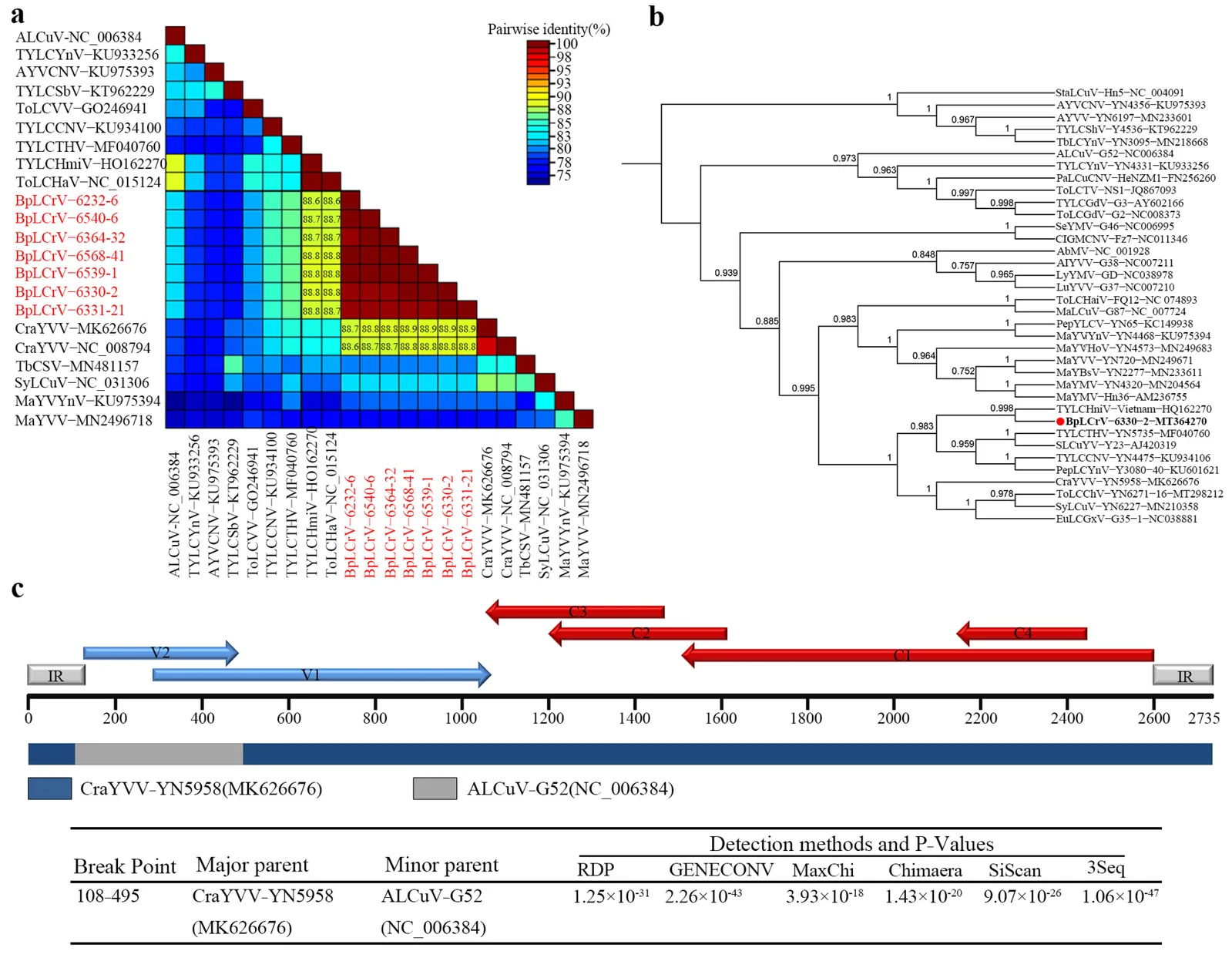
Identification of a novel begomovirus infecting *Bidens pilosa*, *Crassocephalum crepidioides*, tomato, common bean, and pepper plants in Yunnan, China. (**a**) Sequence Demarcation Tool-based pairwise sequence comparisons. The novel isolates of BpLCrV are marked with red font. (**b**) Phylogenetic dendrograms based upon alignments of the begomovirus DNA-A identified in this study with other selected species. The present isolates of BpLCrV-6330-2 are marked with red circles. The phylogenetic tree was constructed using the Maximum Likelihood (ML) method in MEGA X based on the GTR + G + I model, of which the bootstrap analysis had 1000 replicates. (**c**) Analysis of the recombination of BpLCrV-6330-2. A linear genome map of BpLCrV-6330-2 is shown to indicate the positions of possible recombination breakpoints (1740–2691 nt). The intergenic region (IR; gray bar) and genes (with the position and their orientation indicated with arrows: blue for virion-sense and red for complementary-sense) are shown. Putative parental viruses for this recombinant and the algorithms supporting these data, with their average *p*-values, are also listed.

The results of both BLAST and SDT (Figure 2a) showed that these sequences share the highest nucleotide identity (88.73–88.95%) with the known isolate of CraYVV-YN5958 (MK626676). Based on the recommended criteria, a 91% identity as a demarcation threshold for defining new begomovirus species [23], these isolates were therefore classified as a new begomovirus, named bidens pilosa leaf crumple virus (BpLCrV).

The results of pairwise sequence comparisons showed that the sequences of BpLCrV revealed 99.63 to 100.00% identity with each other (Table S4). Based on a sequence identity threshold of 94% for strain demarcation [23], these twelve clones are the same strain of BpLCrV. Thus, the sequence of the BpLCrV-6330-2 (MT364270) isolate was selected for further multiple alignments. The results showed that the BpLCrV-6330-2 sequence can be divided into two parts (Table S5). Part 1 includes the IR, *V1* (CP), *C1* (Rep), and *C4* genes. The predicted amino acid sequences of the CP, Rep, and C4 proteins show the highest identity with CraYVV-YN5958 (93.8%, 96.1%, and 94.9%, respectively). Part 2 includes the *V2*, *C2* (TrAP), and *C3* (REn) genes. The predicted amino acid sequences of the V2, TrAP, and REn proteins show the highest identity with ToLCHaV or TYLCHniV (94.0%, 88.1%, and 88.1%, respectively). Consistently, the sequence from 1386 to 57 nt of BpLCrV-6330-2 shares 95.5% sequence identity with CraYVV-YN5958, but the sequence from 1083 to 1371 nt of BpLCrV-6330-2 shows only 68.9% sequence identity with CraYVV-YN5958, aligning with the highest amino acid sequence identity between the Rep and C4 proteins of BpLCrV-6330-2 and CraYVV-YN5958 but low amino acid sequence identity (72.6%) with the REn protein (Table S5). Thus, it is very likely that BpLCrV-6330-2 originated from recombination between CraYVV-YN5958 and other begomoviruses.

### 3.3. Phylogenetic and Recombination Analysis

The phylogenetic analysis showed that BpLCrV is clustered with PepLCYnV, TYLCCNV, SLCuYV, TYLCTHV, and TYLCHniV in a major clade, and formed a subclade with TYLCHniV (Figure 2b), indicating that BpLCrV is closely related to the TYLCHniV species. RDP analysis revealed a strong recombination event in isolate BpLCrV-6330-2, as supported by six different methods with a high degree of confidence (Figure 2c). The recombination breakpoint was located between nucleotides 108 and 495 (spanning 387 nt), encompassing the complete *V2* gene region (Figure 2c). The major parent of BpLCrV-6330-2 was predicted to be CraYVV-YN5958 (MK626676), while the minor parent was ALCuV-G52 (NC_006384). The related contigs of CraYVV-YN5958 were 2348 nt in length and shared 89.8% sequence identity with BpLCrV-6330-2. However, as shown in the previous multiple alignment analysis, the region from nucleotide 1386 to 57 (spanning 1406 nt) of BpLCrV-6330-2 exhibited 95.5% sequence identity with CraYVV-YN5958. In addition, the related contigs of ALCuV were 387 nt in length and showed 92.8% identity with BpLCrV-6330-2.

### 3.4. Infectivity and Symptoms Induced by YN6330-2

**Figure 3:**
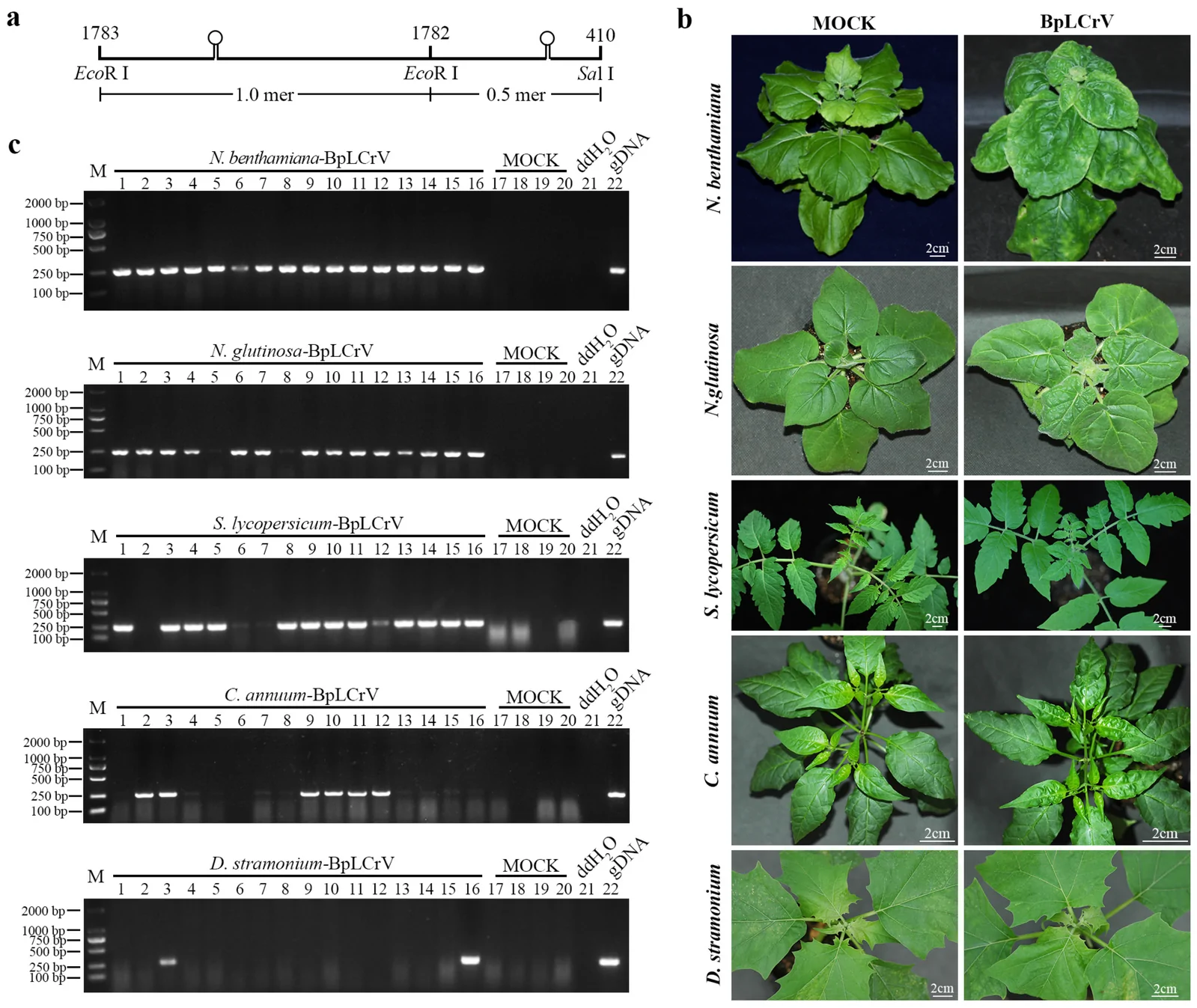
Infectivity and pathogenicity of the infectious clone of BpLCrV. (**a**) Strategies for the construction of the infectious clones of BpLCrV; 1.5 mer tandem repeats of BpLCrV DNA A were constructed for the plant binary vector pBinPLUS. The EcoRI and SalI used for the construction of the infectious clone of BpLCrV are shown. The circles represent stem-loop structures that contain the conserved 5′-TAATATT↓AC-3′ sequence of the BpLCrV DNA A. (**b**) Analysis of the infectivity and pathogenicity of the BpLCrV infectious clone. Symptoms induced by BpLCrV in *N. benthamiana* at 21 dpi, *N. glutinosa* at 21 dpi, tomato (*S. lycopersicum*) at 24 dpi, pepper (*C. annuum*) at 24 dpi, and *D. stramonium* at 23 dpi. Bar = 2 cm. (**c**) PCR detection of BpLCrV viral DNA from plants infected with BpLCrV or buffer (MOCK). Systemic leaves collected from plants at 24 dpi were used for PCR detection. Lane M: DL 2000 marker; Lane 1–16: infected plants; Lanes 17–20: MOCK-inoculated control; Lanes 21: blank control (sterile water); Lane 22: positive control (plasmid control).

To investigate the infectivity of this novel begomovirus, an infectious clone of BpLCrV-6330-2 was constructed (Figure 3a) and agroinfiltrated to 16 plants each of *N. benthamiana*, *N. glutinosa*, tomato, pepper, and *D. stramonium* (Figure 3). In *N. benthamiana*, all infiltrated plants exhibited obvious downward leaf curling and dwarfing at 21 days post-infiltration (dpi) (Figure 3b,c). In *N. glutinosa*, similar symptoms to *N. benthamiana* were also observed at 21 dpi in 14 plants infected with BpLCrV (Figure 3b,c). In tomato (*S. lycopersicum*), mild downward leaf curling and dwarfing symptoms were observed at 24 dpi in 13 plants infected with BpLCrV (Figure 3b,c). In pepper (*C. annuum*), leaf crinkling and stunting were observed at 24 dpi in six plants infected with BpLCrV (Figure 3b,c). In *D. stramonium*, mild downward leaf curling developed at 23 dpi, but only two plants were successfully infected by BpLCrV and exhibited clear symptoms (Figure 3b,c). Therefore, a BpLCrV infectious clone was developed to infect and cause disease symptoms in different host plants, which also validated the virulence of the new begomovirus to plants.

## 4. Discussion

*Bidens pilosa* is a widely distributed weed species and used globally to treat diseases associated with immune response disorders [24]. Weeds play an important role in the epidemiology of begomoviruses, serving both as alternative hosts and as “melting pots” for recombination and satellite exchange [10]. In this study, a novel monopartite begomovirus was identified from weeds of *Bidens pilosa* and *Crassocephalum crepidioides* in field conditions. A previous report showed that *Bidens pilosa* cannot be infected by begomovirus TYLCV under experimental conditions [25]. Our results indicate that weed *Bidens pilosa* serves as an alternative host of begomovirus in field conditions. To the best of our knowledge, this is the first report of *Bidens pilosa* as the host of begomovirus.

Previous reports showed that most weed begomoviruses often failed to infect crops [14]. However, this study showed that tomato, common bean, and pepper crops are also natural hosts of BpLCrV. In addition, infectivity experiments showed that BpLCrV can infect *N. benthamiana*, *N. glutinosa*, tomato, pepper, and *D. stramonium*, causing leaf curling and dwarfing symptoms. These findings demonstrate that this novel begomovirus BpLCrV can infect not only weeds but also crops. Nevertheless, it is worth noting that the symptoms of tomato plants in our infectivity tests (Figure 3b) were not as severe as those collected in fields (Figure 1a). This is most likely attributable to the fact that the tomato plants in fields were infected not only by BpLCrV but also betasatellites, whereas our infectivity assay only employed inoculation with BpLCrV. In addition, because seeds of *Bidens pilosa*, *C. crepidioides*, and common bean were unavailable, the infectivity of BpLCrV on these three plant species was not validated in the present study. Therefore, further research is required to confirm whether the symptoms observed in the field (Figure 1a–c,e) were induced by the BpLCrV or BpLCrV associated with betasatellites.

It is well known that *Geminiviridae* shows the highest evolutionary success among plant viruses largely due to inter- and intra-species recombination [2]. Yunnan (south China) is a reservoir of geminiviruses with the greatest diversity of geminiviruses and their satellites [26]. Based on recently published reports, the emergence of new begomovirus species by recombination is increasing in Yunnan, such as tomato leaf curl Yunnan virus (TLCYnV) [27], pepper yellow leaf curl virus (PepYLCV) [28], tomato yellow leaf curl Shuangbai virus (TYLCSbV) [29], and tomato yellow leaf curl Chuxiong virus (TYLCCxV) [22]. In this study, we also found that BpLCrV likely originated from the recombination of CraYVV and ALCuV. These findings also further hint that a greater diversity of begomoviruses is likely to result in the evolution of more new species through recombination. Previous studies showed that new species that emerged through recombination usually possessed high pathogenicity, a broader host range, or higher transmission efficiency compared with parental viruses [4,27,29]. Further research should be conducted to explore the pathogenicity, host range, and transmission efficiency of BpLCrV.

## 5. Conclusions

In summary, a novel monopartite begomovirus (BpLCrV), likely originating from the recombination between CraYVV-YN5958 and ALCuV-G52, was identified from tomato, common bean, *Bidens pilosa*, pepper, and *Crassocephalum crepidioides* plants in Yunnan. A BpLCrV infectious clone was further developed to infect species of *N. benthamiana*, *N. glutinosa*, tomato, pepper, and *D. stramonium* and to validate the pathogenicity of the new begomovirus. Our findings provide further support that weeds could play an important role in the epidemiology and evolution of begomoviruses, and recombination might be a dominant force to evolve new geminiviruses.

## Data Availability

The original contributions presented in this study are included in the article and the Supplementary Materials. Further inquiries can be directed to the corresponding author.

## Supplementary Materials

The following supporting information can be downloaded at https://www.mdpi.com/article/10.3390/v18080876/s1. Table S1: Origins of field-collected leaf samples with typical symptoms of begomovirus infection; Table S2: BpLCrV genomic features identified from multiple hosts; Table S3: Percent identities between the complete DNA-A of BpLCrV isolates; Table S4: Nucleotide and amino acid identities of isolate BpLCrV-6330-2 (MT364270) with other begomoviruses; Table S5: Begomovirus sequences used for phylogenetic tree construction and recombination analysis; Table S6: Begomovirus sequences used for phylogenetic trees construction and recombination analysis; Figure S1: PCR amplification of begomovirus DNA-A, DNA-B and associated betasatellite or alphasatellite with actin as a control.
