# Supplementary material for: Molecular Characterization of a Novel Monopartite Begomovirus Infecting Weeds and Important Crops in Yunnan, China
Source: Viruses. 2026 Aug 11;18(8):876. doi: 10.3390/v18080876 (PMC13517734; doi:10.3390/v18080876)
Supplement: Supplementary file 1 [file viruses-18-00876-s001.zip › viruses-4439829-supplementary.pdf]

Supplementary

Supplementary Table S1. Origins of field collected leaf samples with typical symptoms of begomovirus infection.

Samples	Host	Locality	Date	Symptoms
YN6232	Tomato	Yunnan: Chuxiong	Nov. 2017	Leaf curling and stunted growth
YN6330	*Bidens pilosa*	Yunnan: Yuanyang	Dec. 2017	Leaf crumple and stunted growth
YN6331	Pepper	Yunnan: Yuanyang	Dec. 2017	Leaf curling and stunted growth
YN6364	*Crassocephalum crepidioides*	Yunnan: Xishuangbanna	Dec. 2017	Vein yellowing and stunted growth
YN6539	Tomato	Yunnan: Chuxiong	Sep. 2018	Leaf curling, small and yellowing, stunted growth
YN6540	Tomato	Yunnan: Chuxiong	Sep. 2018	Leaf curling, small and yellowing, stunted growth
YN6568	Common bean	Yunnan: Chuxiong	Sep. 2018	Leaf curling, small leaves and vein enation

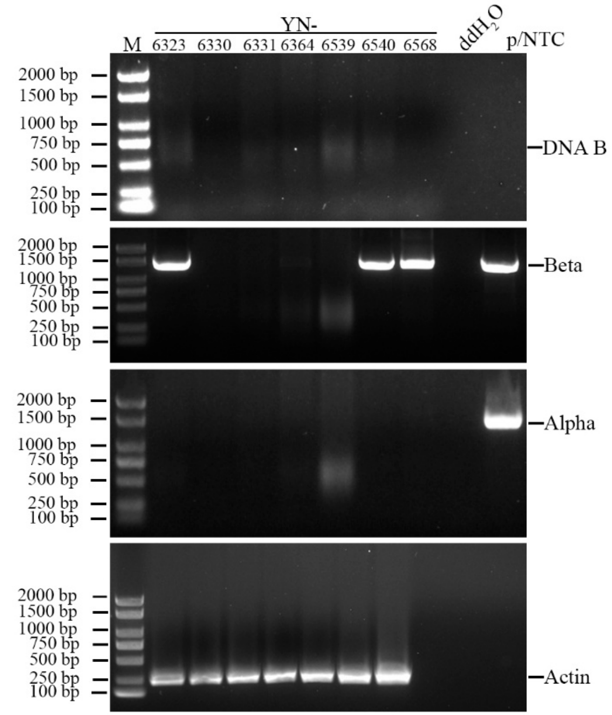

Figure S1. PCR amplification of begomovirus DNA-A, DNA-B and associated betasatellite or alphasatellite with actin as a control. M: DL 2 000 marker; ddH_2O: Blank control; P: Plasmid control; NTC: no-template control. The plasmid served as the positive control in the PCR amplification of betasatellite and alphasatellite, whereas DNA-B and actin lacked a positive control. The line indicates the target band.

Supplementary Table S2 Betasatellite genomic features identified from this study.

Clones	Samples	Accession No.	Total/nt	βC1/nt	SCR/nt	A-rich	
						Location/nt	A/%
YN6232-12	YN6232	PZ761367	1351	196-552	1267-14	756-961	58.25
YN6232-15	YN6232	PZ761368	1346	212-568	1262-14	781-998	66.94
YN6540-56	YN6540	PZ761371	1343	211-567	1259-14	780-990	67.27
YN6540-57	YN6540	PZ761372	1342	212-568	1258-14	765-998	66.67%
YN6568-1	YN6568	PZ761373	1349	196-552	1265-14	772-968	58.18%

Supplementary Table S3 BpLCrV genomic features identified from multiple hosts.

Clones	Samples	Accession No.	Total nt	IR	V1	V2	C1	C2	C3	C4
YN6232-6	YN6232	MT364269	2735	2599-130	291-1064	131-481	1513-2598	1206-1610	1061-1465	2148-2441
YN6330-2	YN6330	MT364270	2735	2599-130	291-1064	131-481	1513-2598	1206-1610	1061-1465	2148-2441
YN6330-5	YN6330	MT364271	2735	2599-130	291-1064	131-481	1513-2598	1206-1610	1061-1465	2148-2441
YN6331-21	YN6331	MT364273	2735	2599-130	291-1064	131-481	1513-2598	1206-1610	1061-1465	2148-2441
YN6331-25	YN6331	MT367575	2735	2599-130	291-1064	131-481	1513-2598	1206-1610	1061-1465	2148-2441
YN6364-32	YN6364	MT367576	2735	2599-130	291-1064	131-481	1513-2598	1206-1610	1061-1465	2148-2441

YN6364-35	YN6364	MT367577	2735	2599-130	291-1064	131-481	1513-2598	1206-1610	1061-1465	2148-2441
YN6539-1	YN6539	MT373110	2735	2599-130	291-1064	131-481	1513-2598	1206-1610	1061-1465	2148-2441
YN6540-6	YN6540	MT373111	2735	2599-130	291-1064	131-481	1513-2598	1206-1610	1061-1465	2148-2441
YN6540-10	YN6540	MT373112	2735	2599-130	291-1064	131-481	1513-2598	1206-1610	1061-1465	2148-2441
YN6568-41	YN6568	MT373116	2735	2599-130	291-1064	131-481	1513-2598	1206-1610	1061-1465	2148-2441
YN6568-45	YN6568	MT373117	2735	2599-130	291-1064	131-481	1513-2598	1206-1610	1061-1465	2148-2441

Supplementary Table S4 Percent identities between the complete DNA-A of BpLCrV isolates.

**BpLCrV-YN**	**6232-6**	**6330-2**	**6330-5**	**6331-21**	**6331-25**	**6364-32**	**6364-35**	**6539-1**	**6540-6**	**6540-10**	**6568-41**	**6568-45**
**6232-6**	-	99.67%	100.00%	99.63%	99.63%	99.74%	99.74%	99.85%	99.82%	99.85%	99.85%	99.82%
**6330-2**	99.67%	-	100.00%	99.96%	99.96%	99.71%	99.71%	99.82%	99.78%	99.82%	99.82%	99.78%
**6330-5**	99.67%	100.00%	-	99.93%	99.96%	99.71%	99.71%	99.82%	99.78%	99.82%	99.82%	99.78%
**6331-21**	99.63%	99.96%	99.96%	-	99.93%	99.67%	99.74%	99.78%	99.74%	99.78%	99.78%	99.74%
**6331-25**	99.63%	99.96%	99.96%	99.93%	-	99.67%	99.67%	99.78%	99.74%	99.78%	99.78%	99.74%
**6364-32**	99.74%	99.71%	99.71%	99.67%	99.67%	-	99.85%	99.89%	99.85%	99.89%	99.89%	99.85%
**6364-35**	99.74%	99.71%	99.71%	99.74%	99.67%	99.85%	-	99.89%	99.85%	99.89%	99.89%	99.85%
**6539-1**	99.85%	99.71%	99.71%	99.74%	99.67%	99.85%	99.89%	-	99.96%	100.00%	100.00%	99.96%
**6540-6**	99.82%	99.78%	99.78%	99.74%	99.74%	99.85%	99.85%	99.96%	-	99.96%	99.96%	99.93%
**6540-10**	99.85%	99.82%	99.82%	99.78%	99.78%	99.89%	99.89%	100.00%	99.96%	-	100.00%	99.96%
**6568-41**	99.85%	99.82%	99.82%	99.78%	99.78%	99.89%	99.89%	100.00%	99.96%	100.00%	-	99.96%

**6568-45**	99.82%	99.78%	99.78%	99.74%	99.85%	99.85%	99.96%	99.96%	99.93%	99.96%	99.96%	-

Supplementary Table S5 Nucleotide and amino acid identities of isolate BpLCrV-6330-2 (MT364270) with other begomoviruses

Accession No.	Isolate	Viruses	Total	IR	V1		V2		C1		C2		C3		C4		C5	
			Nt	Nt	Nt	AA	Nt	AA	Nt	AA	Nt	AA	Nt	AA	Nt	AA	Nt	AA
MK626676	YN5958	CraYVV	**88.7**	**90.3**	**86.8**	**93.8**	83.3	81.0	**95.6**	**96.1**	88.4	80.7	76.5	72.6	**97.3**	**94.9**	-	-
NC_008794	Jinghong	CraYVV	88.6	90.3	86.6	93.4	83.3	78.4	95.5	95.9	88.4	80.7	76.5	72.6	96.3	91.8	-	-
NC_015124	tomato	ToLCHaV	88.1	88.5	79.7	85.3	94.0	94.0	93	93.6	92.3	88.1	90.1	88.1	93.2	85.7	71.1	44.0
HQ162270	Vietnam	TYLCHniV	88.1	88.5	79.7	85.3	94.0	94.0	93	93.6	92.3	88.1	90.1	88.1	93.2	85.7	71.1	44.0
GU723740	J8-2	TYLCTHV	86.1	46.7	83.5	93.0	75.5	70.8	90.4	90.3	88.4	81.5	85.2	80.7	95.2	87.8	88.3	74.0
KU934106	YN4475	TYLCCNV	85.3	83.9	75.7	80.9	86.8	85.3	91.8	94.2	90.4	83.0	82.0	77.8	94.9	87.8	-	-
KU934100	YN4508	TYLCCNV	85.3	82.7	75.2	80.5	85.3	81.9	91.9	92.8	91.4	84.4	83.0	78.5	95.2	86.7	-	-
MW661191	YN149	TYLCCNV	84.1	79.8	75.7	79.4	82.8	81.9	89.8	89.0	91.6	86.7	86.2	84.4	91.8	81.6	72.0	46.0
NC_021205	OX2	TYLCCNV	83.4	79.4	89.1	96.1	90.3	89.7	85.3	84.5	72.8	61.5	70.9	63.0	91.5	79.6	88.7	72.0
MN210358	YN6227	SyLCuV	82.3	74.5	87.3	94.9	83.0	77.6	83.3	84.8	80.7	69.6	74.6	70.4	90.1	79.6	90.0	75.0
AJ811439	G7	PaLCuCNV	82.1	90.3	79.3	85.3	93.2	90.6	84.7	86.2	77.5	66.7	78.8	71.1	93.2	84.7	-	-
PP265534	czraoping	PaLCuCNV	81.8	79.4	79.2	85.3	94.0	93.2	83.3	84.5	78.5	68.9	78.5	72.6	91.8	83.7	71.6	44.0
NC_006384	G52	ALCuV	81.8	79.5	78.8	85.3	92.6	89.7	79.2	79.2	90.6	83.7	89.1	86.7	71.8	42.3	71.1	42.7
GQ246941	Haiphong 23	ToLCVV	80.7	76.3	78.7	84.9	91.5	95.7	86.0	85.9	74.3	65.2	71.1	62.2	90.8	81.6	69.3	37.3

MN481157	YN5438	TbCSV	80.2	75.7	75.9	78.6	83.6	79.3	86.1	86.2	78.0	66.7	73.8	65.2	91.5	81.6	-	-
KF150142	FQ12	ToLCHnV	80.0	72.1	87.1	96.1	90.0	89.7	79.2	79.8	73.6	63.7	71.9	62.2	72.2	42.3	83.3	62.0
MN218668	YN3095	TbLCYnV	79.3	79.9	86.9	94.9	83.0	78.4	77.3	78.5	77.0	68.1	78.0	71.9	71.8	42.3	88.0	74.0
MN249683	YN4573	MaYVHoV	77.7	65.7	86.4	93.4	74.4	63.8	77.9	79.6	70.9	61.2	69.6	65.9	74.5	48.0	89.0	71.0

Supplementary Table S6 Begomovirus sequences used for phylogenetic tree construction and recombination analysis

GenBank acession number	Begomovirus	Acronyms	Function
NC_004091	stachytarpheta leaf curl virus-Hn5	StaLCuV-Hn5	used for phylogenetic trees construction
KU975393	ageratum yellow vein China virus-YN4356	AYVCNV-YN4356	
MN233601	ageratum yellow vein virus-YN6197	AYVV-YN6197	
KT962229	tomato yellow leaf curl Shuangbai virus-Y4536	TYLCShV-Y4536	
MN218668	tobacco leaf curl Yunnan virus-YN3095	TbLCYnV-YN3095	
NC_006384	ageratum leaf curl virus -G52	ALCuV-G52	
KU933256	tomato yellow leaf curl Yunnan virus-YN4331	TYLCYnV-YN4331	
FN256260	papaya leaf curl China virus-HeNZM1	PaLCuCNV-HeNZM1	
JQ867093	tomato leaf curl Taiwan virus-NS1	ToLCTV-NS1	
AY602166	tomato yellow leaf curl Guangdong virus-G3	TYLCGdV-G3	
NC_008373	tomato yellow leaf curl Guangdong virus-G2	ToLCGdV-G2	
NC006995	senecio yellow mosaic virus -G46	SeYMV-G46	
NC011346	clerodendrum golden mosaic China virus-Fz7	CIGMCNV-Fz7	
NC_001928	abutilon mosaic virus	AbMV	
NC007211	alternanthera yellow veinvirus-G38	AIYVV-G38	

NC038978	lycianthes yellow mosaic virus-GD	LyYMV-GD	
NC007210	ludwigia yellow vein virus-G37	LuYVV-G37	
NC_074893	tomato leaf curl Hainan virus-FQ12	ToLCHaiV-FQ12	
NC_007724	malvastrum leaf curl virus-G87	MaLCuV-G87	
KC149938	pepper yellow leaf curl virus-YN65	PepYLCV-YN65	
KU975394	malvastrum yellow vein Yunnan virus-YN4468	MaYVYnV-YN4468	
MN249683	malvastrum yellow vein Honghe virus-YN4573	MaYVHoV-YN4573	
MN249671	malvastrum yellow vein virus-YN720	MaYVV-YN720	
MN233611	malvastrum yellow vein Baoshan virus -YN2277	MaYBsV-YN2277	
MN204564	malvastrum yellow mosaic virus-YN4320	MaYMV-YN4320	
AM236755	malvastrum yellow mosaic virus-Hn36	MaYMV-Hn36	
HQ162270	tomato leaf curl Hanoi virus-Vietnam	TYLCHniV-Vietnam	
**MT364270**	**bidens pilosa leaf crumple virus -6330-2**	**BpLCrV-6330-2**	
MF040760	tomato yellow leaf curl Thailand virus-YN5735	TYLCTHV-YN5735	
AJ420319	squash leaf curl Yunnan virus-Y23	SLCuYV-Y23	
KU934106	tomato yellow leaf curl China virus-YN4475	TYLCCNV-YN4475	
KU601621	pepper leaf curl Yunnan virus-Y3080-40	PepLCYnV-Y3080-40	
MK626676	crassocephalum yellow vein virus-YN5958	CraYVV-YN5958	
MT298212	tomato leaf curl Chuxiong virus-YN6271-16	ToLCChV-YN6271-16	
MN210358	synedrella leaf curl virus-YN6227	SyLCuV-YN6227	
NC038881	euphorbia leaf curl Guangxi virus-G35-1	EuLCGxV-G35-1	
GU723740	tomato yellow leaf curl Thailand virus-J8-2	TYLCTHV-J8-2	
**MT364270**	**bidens pilosa leaf crumple virus -6330-2**	**BpLCrV-6330-2**	used for recombination analysis
HQ162270	tomato leaf curl Hanoi virus-Vietnam	TYLCHniV-Vietnam	
KU934100	tomato yellow leaf curl China virus-YN4508	TYLCCNV-YN4508	
KU934106	tomato yellow leaf curl China virus-YN4475	TYLCCNV-YN4475	

MW661191	tomato yellow leaf curl China virus-YN149	TYLCCNV-YN149
NC_021205	tomato yellow leaf curl China virus-OX2	TYLCCNV-OX2
KF150142	tomato leaf curl Hainan virus-FQ12	ToLCHnV-FQ12
NC_015124	tomato leaf curl Hanoi virus-tomato	ToLCHaV-tomato
MN218668	tobacco leaf curl Yunnan virus-YN3095	TbLCYnV-YN3095
MN481157	tobacco curly shoot virus-YN5438	TbCSV-YN5438
MN210358	synedrella leaf curl virus-YN6227	SyLCuV-YN6227
AJ811439	papaya leaf curl China virus-G7	PaLCuCNV-G7
PP265534	papaya leaf curl China virus-czraoping	PaLCuCNV-czraoping
MN249683	malvastrum yellow vein Honghe virus-YN4573	MaYVHoV-YN4573
MK626676	crassocephalum yellow vein virus-YN5958	CraYVV-YN5958
NC_008794	crassocephalum yellow vein virus-Jinghong	CraYVV-Jinghong
NC_006384	ageratum leaf curl virus-G52	ALCuV-G52
